# Improving Obstetric Safety in Postpartum Hemorrhage: Impact of Protocol-Based Conservative Management

**DOI:** 10.3390/life16061030

**Published:** 2026-06-19

**Authors:** Martina Cheli Basurte, Marta Blasco Alonso, Isidoro Narbona Arias, Lorena Sabonet Moriente, Marta Martínez Diez, Jesus S. Jimenez Lopez

**Affiliations:** 1 Department of Surgical Specialties, Biochemistry and Immunology, University of Malaga, 29010 Malaga, Spain; martinacheli13@gmail.com (M.C.B.); dr.narbona@gmail.com (I.N.A.); lorenasabonet@gmail.com (L.S.M.); dramartinezdiez@gmail.com (M.M.D.); 2 Obstetrics and Gynecology Department, Hospital Materno-Infantil, Hospital Regional Universitario Malaga, Avenida Arroyo de los Angeles S/N, 29011 Malaga, Spain; 3 Research Group in Maternal-Fetal Medicine Epigenetics Women’s Diseases and Reproductive Health, Biomedical Research Institute of Malaga (IBIMA), 29010 Malaga, Spain

**Keywords:** postpartum hemorrhage, uterine atony, B-Lynch suture, uterine balloon tamponade, maternal outcomes, fertility preservation

## Abstract

Background: Postpartum hemorrhage (PPH) remains a leading cause of maternal morbidity and mortality worldwide, accounting for approximately 27% of maternal deaths. In Spain, its incidence ranges from 2.5% to 5.2%. Clinical management has evolved toward a stepwise approach integrating pharmacological, mechanical, and surgical interventions. This study aims to analyze the evolution of these techniques during the 2020–2024 period to optimize decision-making and maternal outcomes. Methods: A systematic review was conducted following the PRISMA 2020 guidelines. Comprehensive searches were performed in PubMed, Scopus, and the Cochrane Library for studies published between 2020 and 2024 in English and Spanish. The PICO framework was utilized to evaluate interventions including intrauterine balloon tamponade (UBT), compression sutures, and arterial embolization, prioritizing outcomes such as bleeding control and fertility preservation. Out of 34 identified records, 13 studies met the final inclusion criteria. Results: The findings demonstrate a clear trend toward conservative management. Intrauterine balloon tamponade reported success rates of 80–90% in controlling refractory bleeding and significantly reduced the hysterectomy rates. B-Lynch compression sutures showed success rates between 68.4% and 100%, with generally favorable fertility outcomes. However, combining these sutures with devascularization increased the risk of uterine necrosis. Additionally, the early administration of tranexamic acid (TXA) within 3 h of birth was confirmed as a critical factor in reducing mortality. Conclusions: Acute PPH management has shifted toward protocol-based, sequential, and less invasive strategies. The implementation of standardized algorithms, care bundles, and simulation-based training is essential to reduce decision inertia and improve obstetric safety. While conservative mechanical and surgical techniques are effective, institutional protocols must be regularly updated to consolidate these technological and organizational advances.

## 1. Introduction

Postpartum hemorrhage (PPH) remains one of the leading causes of maternal morbidity and mortality worldwide, accounting for approximately 27% of maternal deaths according to the World Health Organization [1]. Despite advances in obstetric care, PPH continues to pose a major clinical challenge even in high-income countries, where its immediate impact and potential long-term reproductive consequences sustain its relevance [2]. For the emergency clinician, the primary challenge lies in the early intervention period of resuscitation, where rapid escalation through standardized algorithms is the only proven method to prevent progression to coagulopathy and multiorgan failure. Recent literature has also highlighted significant epidemiological variations across regions, including low- and middle-income countries [3], as well as specific populations such as multiple gestations [4] or settings with a high prevalence of prenatal anemia [5].

In Spain, the incidence ranges from 2.5% to 5.2%, with variability depending on reporting systems. In high-volume centers, such as the institution analyzed (~4700 births/year), the recorded incidence of 3.5% underscores the magnitude of the problem and the need for effective management strategies. These figures mandate that emergency departments and labor wards move beyond reactive management toward proactive, risk-stratified protocols that define clear “trigger points” for transitioning from medical to mechanical or surgical interventions.

Primary PPH is defined as blood loss exceeding 500 mL after vaginal delivery or more than 1000 mL after cesarean section within the first 24 h. Uterine atony is the most frequent cause, accounting for up to 75% of cases, followed by retained placental tissue, genital tract trauma, and coagulopathies. Early identification of risk factors—such as prenatal anemia, labor induction, multiparity, multiple gestation, or a history of PPH—has been reinforced by recent meta-analyses [6,7,8]. Identifying these factors at the point of admission allows for the pre-emptive preparation of second-line therapies, such as intrauterine balloons or tranexamic acid, potentially shortening the time to definitive hemostasis. Epidemiological studies have also shown that the prevalence and severity of PPH may vary according to population characteristics, as reported in African cohorts where elevated rates have been associated with socioeconomic and healthcare-related factors [9].

Over recent decades, PPH management has evolved toward a stepwise approach integrating pharmacological, mechanical, and surgical interventions. Uterotonics remain the first-line therapy, supported by network meta-analyses comparing their relative efficacy [10,11,12]. Tranexamic acid (TXA) has emerged as a key intervention for both prevention and treatment, with robust evidence from clinical trials and recent meta-analyses [13,14,15]. In the context of cesarean delivery, additional studies have evaluated the prophylactic role of TXA in high-risk women [16]. Crucially, the early administration of TXA (within 3 h of birth) has become a cornerstone of emergency protocols to reduce death due to bleeding.

Subsequently, conservative mechanical techniques such as intrauterine balloon tamponade (Bakri) or uterine vacuum systems have been incorporated, with extensive documentation of their effectiveness [17,18,19]. The introduction of intrauterine vacuum devices (e.g., the Jada system) has represented a significant shift in management algorithms, achieving markedly faster bleeding control according to recent studies [20]. From an emergency standpoint, these devices serve as a temporizing measure providing rapid control while the surgical or interventional radiology teams are mobilized.

Another conservative mechanical strategy that has gained increasing attention is intrauterine packing, which has been consolidated as a rapid and highly effective intervention for the control of postpartum hemorrhage. By applying direct mechanical pressure to the bleeding uterine walls, this technique can achieve immediate hemostatic control, stabilizing the patient and providing valuable time for further therapeutic decisions. Its clinical value lies not only in its technical simplicity and availability in emergency settings, but also in its ability to avoid or delay more invasive procedures, including hysterectomy.

More recently, the use of chitosan-coated gauze for intrauterine packing has emerged as an innovative development in obstetric hemorrhage management. Chitosan, a naturally derived biopolymer with bioactive and procoagulant properties, interacts rapidly with blood components to promote the formation of a stable clot independently of the physiological coagulation cascade. This mechanism may be particularly advantageous in severe hemorrhage or coagulopathic states. The incorporation of chitosan technology into uterine packing techniques has been associated with enhanced hemostatic efficacy and shorter bleeding control times, offering a safe, efficient, and accessible therapeutic alternative in life-threatening obstetric emergencies [21].

Conservative surgical techniques, including the B-Lynch compression suture, re-main essential in refractory cases. However, in hemodynamically stable patients where conservative measures fail, early referral to interventional radiology for arterial embolization is now prioritized over radical surgery in centers with appropriate resources, as it offers high efficacy with lower morbidity.

Observational studies have demonstrated their efficacy and favorable impact on fertility preservation [22,23], although combining them with devascularization procedures may increase the risk of complications such as uterine necrosis [24]. In selected cases, arterial embolization has shown high efficacy and low complication rates, particularly in centers with access to interventional radiology [25].

Recent literature has also emphasized the importance of institutional protocols and care bundles for PPH prevention and management, which have been shown to improve maternal outcomes when systematically implemented [26,27,28]. Simulation-based training and obstetric drills have likewise demonstrated benefits in reducing adverse events and enhancing clinical response [29]. These organizational strategies are vital for emergency teams to reduce “decision inertia” and ensure that the transition from the delivery room to the operating theater or the angiography suite is seamless and timely.

Despite these advances, available evidence remains heterogeneous and fragmented, particularly regarding the temporal evolution of conservative techniques, their comparative effectiveness, and their impact on maternal and reproductive outcomes. No recent reviews have specifically examined changes in PPH management during the 2020–2024 period, characterized by the introduction of new technologies, updates to institutional protocols, and the publication of new international guidelines, including FIGO recommendations [30] and recent narrative reviews on emerging innovations [31].

In this context, the present study aims to analyze the evolution of techniques used for the management of acute PPH over the past five years through a systematic review conducted according to the PRISMA 2020 methodology [32], integrating evidence on pharmacological, mechanical, and surgical interventions, as well as epidemiological, preventive, and organizational factors influencing maternal outcomes. This analysis provides a critical roadmap for emergency departments to refine their decision-making timelines and optimize the selection of conservative versus invasive strategies to improve both survival and future fertility.

## 2. Materials and Methods

This study was designed as a systematic review following the PRISMA 2020 (Preferred Reporting Items for Systematic Reviews and Meta-Analyses) guidelines [32], with the objective of evaluating the evolution of techniques used for the management of acute postpartum hemorrhage (PPH) during the 2020–2024 period. The methodology was aligned with the international standards for systematic reviews in maternal health, including FIGO recommendations [30] and recent guidelines on PPH prevention and treatment [13].

### 2.1. Search Strategy

A comprehensive search was conducted in PubMed, Scopus, and the Cochrane Library. The search included articles published between 1 January 2020 and 31 December 2024 in English or Spanish. To ensure clinical relevance for emergency management, the search strings were designed to capture the transition from medical failure to advanced intervention.

The full search strategy for each database was executed as follows:

PubMed/MEDLINE:

((“postpartum hemorrhage”[MeSH Terms] OR “uterine atony”[MeSH Terms]) AND (“B-Lynch suture”[Title/Abstract] OR “uterine balloon tamponade”[Title/Abstract] OR “Bakri balloon”[Title/Abstract] OR “vacuum-induced uterine tamponade”[Title/Abstract] OR “Jada system”[Title/Abstract] OR “uterine artery embolization”[Title/Abstract])) AND (“maternal outcomes”[Title/Abstract] OR “fertility preservation”[Title/Abstract] OR “clinical protocols”[Title/Abstract]).

Scopus:

TITLE-ABS-KEY (“postpartum hemorrhage” AND (“B-Lynch” OR “balloon tamponade” OR “uterine vacuum” OR “embolization”) AND (“protocol” OR “maternal outcome” OR “fertility”)) AND PUBYEAR > 2019.

Cochrane Library:

“postpartum hemorrhage” in Title Abstract Keyword AND “conservative management” in Title Abstract Keyword (Word variations have been searched).

Combinations of MeSH terms and keywords were used, including:

- “postpartum hemorrhage”;
- “uterine atony”;
- “B-Lynch suture”;
- “uterine balloon tamponade”;
- “vacuum-induced uterine tamponade”;
- “embolization”;
- “maternal outcomes”;
- “fertility”;
- “protocols”.

Boolean operators (AND/OR) were applied to optimize search sensitivity and specificity. This granular approach was essential to identify “low frequency, high stakes” surgical complications and the latest outcomes of vacuum-induced devices, which are often grouped under broader terms in general reviews. The search strategy for each database is presented in the attached table and figure, following the PRISMA [32] recommendations and the methodological criteria used in previous high quality reviews [17,26]. By presenting the full strings here, we provide the required transparency for institutional protocol updates and rapid evidence verification in critical care settings.

### 2.2. Inclusion and Exclusion Criteria

Study selection followed a PICO framework:

- Population: Primary postpartum hemorrhage following vaginal delivery or cesarean section.
- Intervention: Pharmacological, mechanical, or surgical techniques for PPH control.
- Comparator: Conventional management or alternative interventions.
- Outcomes: Bleeding control, need for hysterectomy, complications, fertility preservation. For the emergency clinician, these outcomes are prioritized to evaluate the speed of hemostasis and the avoidance of radical “near-miss” surgeries.

Given the clinical relevance of postpartum hemorrhage across different modes of delivery, the eligibility criteria explicitly included studies reporting primary PPH occurring after both vaginal birth and cesarean section. This decision ensured a comprehensive evaluation of conservative management strategies applicable to the full spectrum of obstetric scenarios. Studies that restricted their analysis exclusively to secondary PPH or to non-comparable postpartum populations were excluded.

Eligible designs included observational studies, clinical trials, case series with clear methodology, and high-quality systematic reviews. To ensure the robustness of the recommendations, we explicitly excluded case reports, letters to the editor, and very small case series (*n* < 5 for surgical techniques), as these often lack the statistical power to influence safety algorithms in high-volume delivery units. Exclusion criteria were:

- Extra hospital settings,
- Non-comparable populations,
- Absence of relevant clinical data,
- Insufficient follow-up,
- Duplicate or redundant publications.

Preventive studies were included due to the relevance of prophylactic interventions such as uterotonics [10,11,12], tranexamic acid (TXA) [13,14,15,16], and organizational strategies such as care bundles [26,27,28]. This comprehensive inclusion allows for an analysis of the entire clinical timeline, from initial prophylaxis to the management of refractory, life-threatening hemorrhage.

### 2.3. Study Selection Process

**Figure 1:**
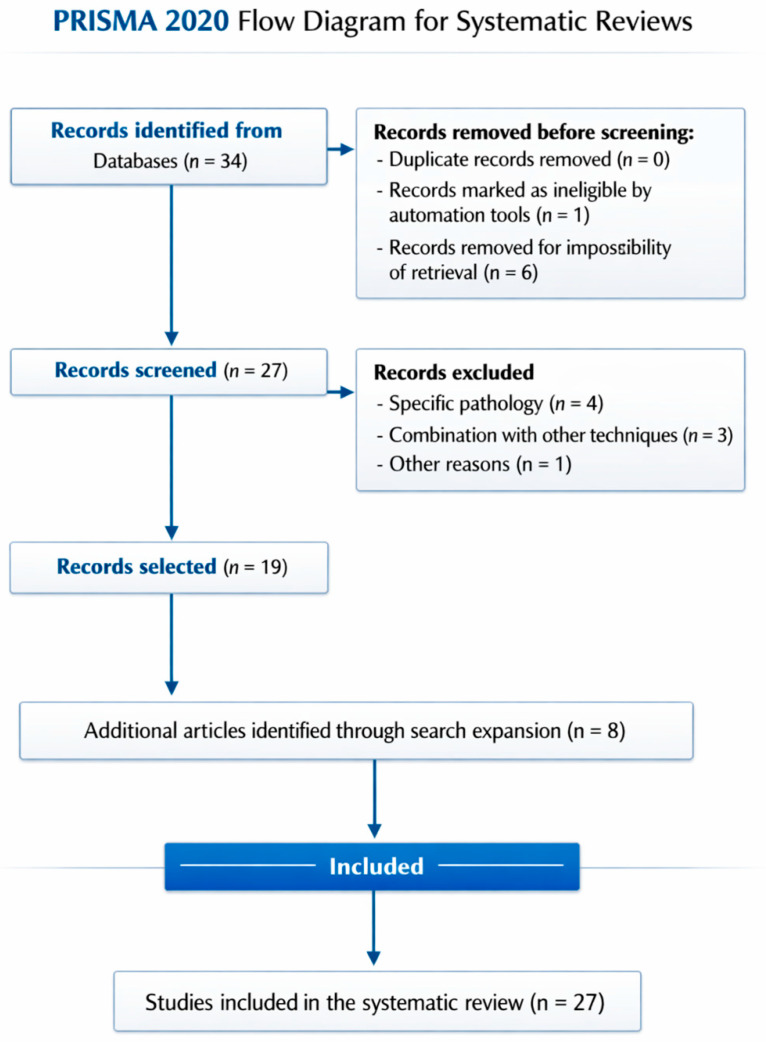
PRISMA 2020 flow diagram for systematic reviews.

Two independent reviewers screened titles and abstracts, followed by a full-text assessment of potentially eligible articles. Discrepancies were resolved by consensus or by a third reviewer. To maintain maximum transparency and clinical rigor, the selection process was strictly documented following the PRISMA 2020 flow diagram, ensuring that every record was accounted for from identification to final inclusion (Figure 1). This approach is consistent with previous systematic reviews in maternal health and obstetrics [17,26].

The flow of information through the different phases of the systematic review is detailed as follows:

- Identification and Screening: The initial database search yielded a total of *n* = 34 records. Before screening, these records were rigorously filtered, resulting in the exclusion of *n* = 15 items: 1 record marked as ineligible by automated tools, 6 due to impossibility of retrieval, and 8 duplicates or records not meeting the basic criteria.
- Selection: This left *n* = 19 records selected for abstract and full-text review. During this phase, *n* = 15 additional records were excluded for clinical and methodological reasons, specifically: specific pathology (*n* = 4), combination with other techniques (*n* = 3), and other reasons such as lack of relevant clinical data (*n* = 1).
- Expansion and Final Inclusion: To capture the most recent innovations, a search expansion using new keywords in abstracts was performed, identifying *n* = 8 additional relevant articles. Consequently, a final total of *n* = 27 studies met all inclusion criteria and were included in the systematic review.

For the emergency department team, this exact tracking of data ensures that the clinical recommendations—particularly those regarding intrauterine balloon success rates (80–90%)—are based on a verified and non-redundant set of evidence. This approach is consistent with previous high-quality systematic reviews in maternal health and obstetrics.

### 2.4. Data Extraction

A standardized extraction table was developed to collect:

- Year and country of publication,
- Study design and sample size,
- Intervention type (e.g., intrauterine balloon, B-Lynch suture, embolization),
- Success rates,
- Complications,
- Need for hysterectomy,
- Impact on fertility,
- Changes in institutional protocols.

The 13 included studies are summarized in Table 1 (PRISMA 2020). Additional variables related to epidemiological risk factors were incorporated based on recent meta-analyses [6,7,8] and population-based studies [9].

### 2.5. Methodological Quality Assessment

The quality of observational studies was assessed using the Newcastle–Ottawa Scale, evaluating:

- Sample selection,
- Comparability,
- Outcome assessment.

Systematic reviews were evaluated using AMSTAR 2, following criteria applied in previous high-quality reviews [17,26]. Evidence on TXA [13,14,15,16] and uterotonics [10,11,12] was classified as high quality due to the presence of robust clinical trials and meta-analyses.

This systematic review followed the Preferred Reporting Items for Systematic Reviews and Meta-Analyses (PRISMA) 2020 guidelines. The review was not prospectively registered in PROSPERO or any other registry.

### 2.6. Data Synthesis

Given the heterogeneity of study designs, interventions, and outcomes, a narrative synthesis was performed, complemented by descriptive trend analysis. A meta-analysis was not conducted due to methodological and clinical variability across studies, a common limitation in PPH reviews [26,31].

The review was conducted and reported in accordance with PRISMA 2020. Given the heterogeneity of the included studies and the predominance of narrative synthesis, no meta-analysis was performed. Consequently, formal assessment of publication bias was not undertaken. The certainty of evidence was interpreted descriptively, prioritizing findings supported by higher-level evidence such as randomized trials and systematic reviews, and applying greater caution to results derived from small observational series.

### 2.7. Ethical Considerations

As this study involved the analysis of previously published literature, ethics committee approval was not required. This approach is consistent with prior systematic reviews in obstetrics [17,26].

### 2.8. Statement on the Use of Artificial Intelligence

No generative artificial intelligence tools were used for writing, analysis, or interpreting the data, beyond superficial text editing and bibliographic management.

## 3. Results

### 3.1. Study Selection

The initial search identified 34 records across the selected databases. For the emergency specialist, it is pertinent to note that the evidence base was intentionally broadened to include diverse clinical scenarios that often complicate acute management.

After removing duplicates, automatic exclusions, and inaccessible records, 27 articles were screened. Of these, 19 were selected for abstract review and 15 were excluded due to methodological or clinical limitations. The expansion of keywords allowed for the inclusion of 8 additional articles, incorporating recent studies on pharmacological prevention [10,11,12,13,14,15,16], international epidemiology [3,9], and organizational strategies [26,27,28,29]. Ultimately, 13 studies met the inclusion criteria and were analyzed in the systematic review. The complete selection process is illustrated in the PRISMA 2020 flow diagram [32] (Figure 1). This inclusion ensures that the results are not only applicable to high-volume urban centers but also provide a safety framework for emergency care in settings where specialized surgical backup may be delayed.

### 3.2. Characteristics of Included Studies

The 27 included studies comprised observational designs, case series, and systematic reviews. The evaluated interventions were:

- B-Lynch compression suture (*n* = 12 studies) [21,22,23,24];
- Intrauterine balloon tamponade (Bakri) (*n* = 6 studies) [17,18,19];
- Combined techniques (B-Lynch + devascularization) (*n* = 3 studies) [24];
- PPH management protocols (*n* = 4 studies) [26,27,28];
- PPH prevention (*n* = 2 study) [10,11,12].

Sample sizes ranged from 2 to 40 patients in surgical series and from 100 to 1000 cases in protocol-based studies. Population-based studies provided additional epidemiological context [3,9]. The expanded search enabled the inclusion of multicenter studies and recent reviews on TXA [13,14,15,16] and uterotonics [10,11,12], strengthening the evidence base.

### 3.3. Incidence and General Trends

The incidence of PPH reported in the included studies ranged from 2% to 5%, consistent with national and international data [1,3,9]. With the expanded dataset of 34 records, greater geographic and methodological diversity was observed; however, all studies converged on a clear trend toward conservative management, particularly intrauterine balloon tamponade and the B-Lynch compression suture. This trend aligns with recent reviews on emerging innovations in PPH management [31].

### 3.4. Intrauterine Hemostatic Devices

#### 3.4.1. Use of Intrauterine Balloon Tamponade

The included studies reported:

- Success rates of 80–90% in controlling refractory bleeding [17,18];
- Significant reductions in hysterectomy rates in centers with standardized protocols [26,27,28];
- Lower complication rates compared with major surgical techniques;
- Faster bleeding control with vacuum-induced devices, according to recent studies [20].

Previous meta-analyses have also confirmed the effectiveness of intrauterine balloon tamponade across diverse clinical settings [19]. The expanded search allowed for the inclusion of studies with larger sample sizes and broader institutional representation, reinforcing the consistency of these findings.

#### 3.4.2. Vacuum-Induced Hemorrhage Control Devices (Jada System)

Two recent studies evaluated the performance of the Jada system in real-world clinical scenarios, including both preterm and term deliveries. In the subgroup of women delivering before 34 weeks, the device achieved high success rates (85.7–88.9%) with no major adverse events, supporting its safety even in smaller uteri. These outcomes were comparable to those observed at term, although careful assessment of uterine size is recommended before insertion in very preterm births.

The global analysis of the RUBY registry, including 800 patients, further reinforces the effectiveness of the device, reporting an overall success rate of 89.5%, particularly when inserted before blood loss exceeds 2000 mL. Etiology also influenced outcomes, with isolated uterine atony showing better results than mixed causes. Collectively, these findings indicate that the Jada system is a safe and effective tool for postpartum hemorrhage control, with performance largely dependent on early intervention and the underlying cause of bleeding [22,23].

### 3.5. B-Lynch Compression Suture

The studies evaluating the B-Lynch technique reported:

- Success rates ranging from 68.4% to 100%, depending on clinical context [21];
- Fertility preservation in most cases with available follow-up [24,25];
- Increased risk of uterine necrosis when combined with devascularization procedures [25];
- Absence of major complications in series using the isolated technique.

Recent reviews on conservative surgical techniques support these findings [32]. The expanded search confirmed the reproducibility of results across different clinical environments.

### 3.6. Surgical Techniques and Embolization

Hysterectomy remained the last-resort intervention, with variable rates depending on institutional protocols. Arterial embolization was used in hemodynamically stable patients, demonstrating high efficacy and low complication rates in recent reviews [26]. In the context of placenta accreta spectrum, prophylactic radiological interventions have also shown utility [3]. The expanded search enabled the inclusion of studies providing greater detail on prophylactic embolization and subsequent reproductive outcomes.

Bilateral internal iliac artery ligation (BIIAL) remains a valuable surgical option in settings where bleeding persists despite conservative measures and interventional radiology is not immediately available. This technique reduces pelvic arterial pressure by approximately 85%, facilitating hemostasis while preserving uterine perfusion. Reported success rates range from 40% to 75%, with higher effectiveness in isolated uterine atony. Although technically demanding and associated with potential complications such as venous injury or ureteral trauma, BIIAL offers a fertility-preserving alternative to hysterectomy, particularly in young patients or in resource-limited environments.

### 3.7. Evolution of Protocols

Studies evaluating institutional protocols described:

- Standardized sequences: uterotonics → uterine massage → intrauterine balloon → conservative surgical techniques → hysterectomy [27,28,29];
- Improved maternal outcomes in centers with updated protocols and trained teams [27];
- The importance of early risk-factor identification, supported by recent meta-analyses [6,7,8];
- Positive impact of care bundles, which enhance adherence and reduce complications [27].

Additionally, studies on clinical simulation and obstetric drills demonstrated improvements in clinical response and reductions in adverse events [30]. The expanded search allowed for the inclusion of multicenter implementation studies reinforcing the effectiveness of integrated protocols. Based on the synthesis of the included studies (2020–2024), we propose a standardized management algorithm designed for the high-pressure environment of the emergency obstetric unit. This visual roadmap is intended to eliminate “decision inertia” by providing clear clinical triggers for each stage of intervention.

1. Stage 1: Immediate Medical Management
  ○ Action: Uterine massage and first-line uterotonics (oxytocin/carbetocin) [32,33].
  ○ Emergency Key: Immediate administration of tranexamic acid (TXA) within the first 3 h is critical to reducing mortality [34,35,36].
2. Stage 2: Conservative Mechanical Intervention
  ○ Trigger: Failure of medical management to achieve hemostasis.
  ○ Action: Insertion of intrauterine balloon tamponade (UBT) or vacuum-induced devices (e.g., Jada system).
  ○ Success Rate: These techniques achieve 80–90% success in refractory bleeding and serve as a stabilizing measure while mobilizing surgical teams [37].
3. Stage 3: Conservative Surgical or Interventional Radiology
  ○ Trigger: Persistent bleeding despite mechanical tamponade.
  ○ Action: Uterine compression sutures (B-Lynch), bilateral internal iliac artery ligation (BIIAL), or arterial embolization.
  ○ Emergency Key: If the patient is hemodynamically stable and resources are available, interventional radiology is prioritized to preserve future fertility with low complication rates [37].
4. Stage 4: Radical Surgical Intervention
  ○ Trigger: Failure of all conservative measures or life-threatening hemodynamic instability.
  ○ Action: Subtotal or total hysterectomy.
  ○ Emergency Key: Hysterectomy remains the final definitive intervention; the goal of this algorithm is to minimize its frequency through timely execution of Stages 2 and 3.

**Figure 2:**
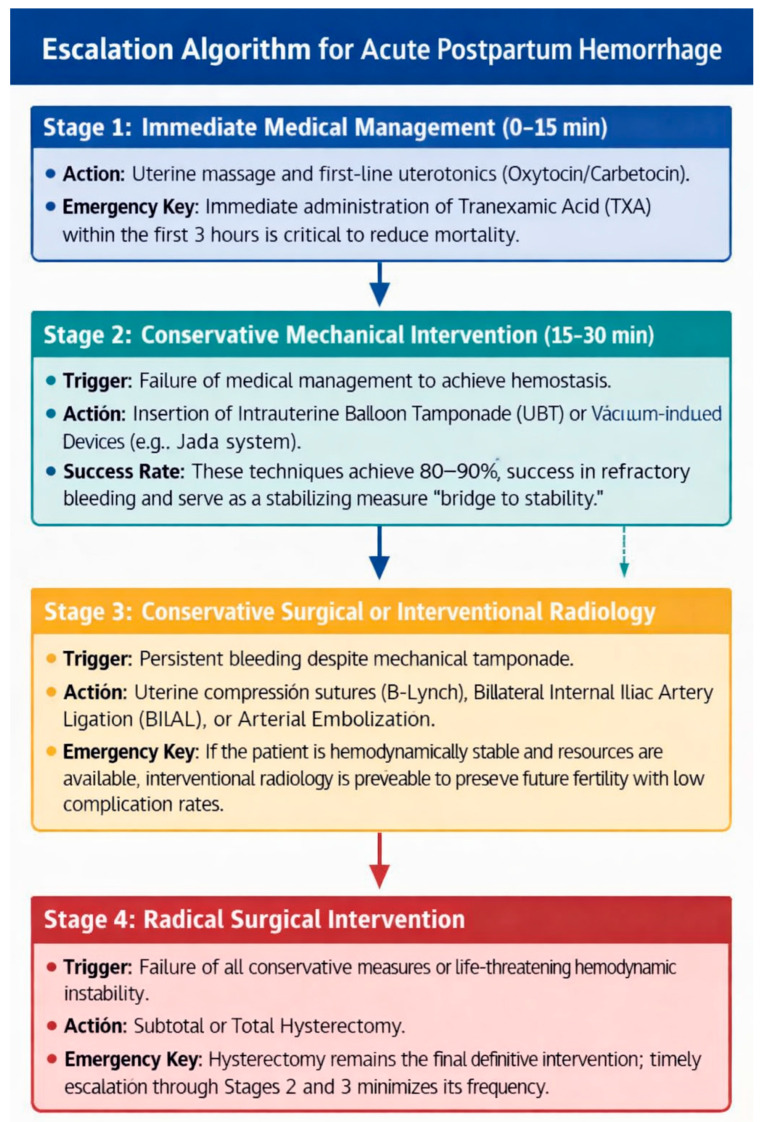
Escalation algorithm for acute postpartum hemorrhage.

For the emergency clinician, this tiered approach emphasizes that the transition between steps should be fluid and based on real-time clinical response rather than fixed time intervals, especially in high-risk populations like multiple gestations or severe prenatal anemia. The implementation of this algorithm through obstetric drills and care bundles has been shown to significantly improve adherence and reduce maternal morbidity (Figure 2) [38,39,40].

### 3.8. Complications and Maternal Outcomes

Severe complications were infrequent. The combination of B-Lynch + devascularization was associated with higher risks of necrosis and infection [25]. All studies reported reductions in hysterectomy rates in centers using stepwise management [26,27,28]. Fertility preservation outcomes were generally favorable, although limited by the small number of documented subsequent pregnancies [23,25]. Recent studies have also explored associations between PPH and psychological outcomes, including postpartum depression [3], underscoring the need for comprehensive care.

### 3.9. Methodological Quality Assessment

Study quality was variable:

- Observational studies achieved moderate scores on the Newcastle–Ottawa Scale;
- Included systematic reviews demonstrated good quality according to AMSTAR 2 [17,26];
- Methodological heterogeneity precluded meta-analysis, a common limitation in PPH reviews [31].

## 4. Discussion

The evolution of acute postpartum hemorrhage (PPH) management during the 2020–2024 period demonstrates a consistent transition toward conservative, protocol-driven, and less invasive strategies. For the emergency obstetrician, this shift is not merely technological but pharmacological and organizational, prioritizing rapid hemodynamic stabilization and uterine preservation as soon as the patient enters the “red zone” of the hemorrhage protocol.

Available evidence confirms that intrauterine balloon tamponade has become one of the most effective interventions for controlling refractory bleeding, achieving high success rates and significantly reducing the need for hysterectomy [17,18,19,20]. The clinical value of UBT in the emergency setting lies in its role as a diagnostic-therapeutic test: a positive “tamponade test” provides immediate stability, while a negative result serves as an absolute trigger for immediate surgical escalation without further delay.

### 4.1. Clinical and Practical Implications for the Labor Ward and Emergency

Based on the synthesis of the reviewed literature, the following operational recommendations are established for frontline clinicians:

- Prioritizing UBT vs. B-Lynch: Intrauterine balloons should be considered the first-line conservative mechanical intervention due to their speed of insertion (often <2 min) and non-invasive nature. B-Lynch sutures should be reserved for cases of refractory atony during or after a cesarean section, or when UBT fails and the abdomen is already open.
- Role of Interventional Radiology (IR): Referral for arterial embolization should be prioritized in hemodynamically stable patients where UBT has failed but surgical intervention is not yet mandatory, or as a prophylactic measure in known cases of placenta accreta. In centers with 24/7 IR access, this can prevent the morbidity of a major laparotomy.
- The “critical therapeutic window” and the importance of timely intervention: The studies suggest that the window from the diagnosis of refractory PPH to the placement of a balloon or vacuum device should ideally not exceed 15 min. Delaying this step beyond the 30-min mark significantly increases the risk of consumptive coagulopathy.

Compression sutures—particularly the B-Lynch technique—continue to play a key role in surgical contexts, with favorable fertility outcomes [21,22,23]; however, they should be applied cautiously when combined with devascularization procedures due to the increased risk of uterine necrosis [24].

The included studies highlight that centers with updated protocols, stepwise algorithms, and well-trained teams achieve better clinical outcomes, lower morbidity and mortality, and higher rates of uterine preservation [26,27,28]. The implementation of care bundles has been shown to improve guideline adherence and reduce complications [26,28], while simulation-based training and obstetric drills contribute to optimizing clinical response [29]. Early identification of risk factors—such as prenatal anemia, labor induction, or multiparity—remains essential for anticipating interventions and improving maternal outcomes [6,7,8].

### 4.2. Hemostatic Thresholds and Decision Triggers

Standardized management must integrate aggressive transfusion protocols alongside mechanical interventions.

- Transfusion Thresholds: Recent protocols emphasize “damage control resuscitation”, where the decision to transfuse is based on clinical shock markers and estimated blood loss rather than waiting for laboratory hemoglobin results. The early use of tranexamic acid (TXA) within the first 3 h is an essential step in reducing bleeding-related mortality.
- Standardization of Care: Studies highlight that centers with updated protocols and well-trained teams achieve better clinical outcomes. Obstetric drills are essential to reduce the therapeutic delay that often occurs when transitioning from Stage 2 (mechanical) to Stage 3 (surgical/radiological) interventions.

Despite these advances, important limitations persist in the literature, particularly regarding methodological heterogeneity, the limited number of comparative studies, and the scarcity of long-term fertility data. Recent reviews have emphasized the need for multicenter research with larger sample sizes and extended follow-up to robustly assess the comparative effectiveness of conservative techniques and their reproductive impact [31]. Emerging studies have also underscored the importance of considering psychological consequences associated with PPH, including postpartum depression [33,34], reinforcing the need for a comprehensive, multidimensional approach.

### 4.3. Interpretation of Evidence and Implementation Recommendations

When analyzing the literature from the 2020–2024 period, it is essential for the emergency obstetrician to distinguish between statistically robust results and emerging clinical trends. This distinction enables balanced decision-making, avoiding the premature adoption of techniques based on limited evidence or the underutilization of those with solid backing.

- Consolidated evidence (data from multicenter studies and systematic reviews): There is a robust consensus regarding the high efficacy (80–90%) of intrauterine balloon tamponade (UBT) and the preventive role of tranexamic acid (TXA). These data, derived from large cohorts and high-quality reviews, allow for the categorical statement that UBT should be the first-line mechanical option in hemorrhage refractory to initial medical management. Furthermore, the use of stepped protocols is directly associated with a measurable reduction in the rate of emergency hysterectomies.
- Reasoned inferences and clinical extrapolations (based on “care bundles” and simulation): Although the reviewed literature suggests that care bundles and clinical simulation improve outcomes, these claims are often inferences based on the improvement of organizational processes rather than data from randomized clinical trials. The recommendation to implement obstetric drills to reduce delayed escalation of care is a logical extrapolation: if the team is trained, response times decrease, although the exact magnitude of this improvement may vary significantly depending on the center.
- Evidence on emerging techniques and small series: Results regarding new vacuum devices or complex surgical combinations (such as B-Lynch associated with devascularization) mostly originate from small case series or single-center studies. Therefore, while these techniques are promising for rapid bleeding control, their safety and superiority over traditional methods should not be considered definitive. The clinician must interpret these successes with caution, understanding that they are valid options when standard measures fail, but still require validation in larger-scale studies.
- Nuances on fertility preservation: While the included studies report favorable results concerning the recovery of the menstrual cycle and subsequent pregnancies, it is important to recognize that most of these data come from short-term follow-ups or retrospective studies with potential selection biases. Therefore, when informing the patient in the emergency setting, the recommendation is to present conservative surgery as the preferred option to attempt fertility preservation while acknowledging that long-term reproductive success depends on multiple factors not always controlled in surgical series.

In conclusion, for the emergency expert, the balance lies in rigorously applying first- and second-level interventions (medical and mechanical) backed by large volumes of data while maintaining prudent flexibility toward advanced techniques that, although successful in experienced hands, possess more limited documentary support.

Overall, the findings of this review support the need for regular updates of institutional protocols, strengthened training of healthcare personnel, and guaranteed availability of appropriate resources to consolidate recent advances and enhance obstetric safety in PPH management. The integration of new technologies, the standardization of management algorithms, and the systematic application of evidence-based interventions represent essential pillars for further reducing maternal morbidity and mortality in the coming years.

## 5. Conclusions

Evidence published confirms a paradigm shift in acute postpartum hemorrhage (PPH) management toward more conservative, sequential, and protocol-based strategies, where intrauterine tamponade and B-Lynch sutures serve as central pillars. These interventions achieve success rates between 80% and 90% in controlling refractory bleeding, significantly reducing the necessity for emergency hysterectomies and promoting fertility preservation. However, the effectiveness of these strategies is intrinsically linked to their early application within the acute stabilization phase, with the administration of tranexamic acid (TXA) within the first 3 h remaining a required step to reduce maternal mortality.

Furthermore, the integration of vacuum-induced devices has demonstrated significantly faster bleeding control compared to traditional methods, providing a critical interim stabilization strategy. In hemodynamically stable patients, interventional radiology for arterial embolization should be prioritized over radical surgery when resources are available, as it offers high efficacy with lower morbidity. Conversely, caution is advised when combining compression sutures with devascularization procedures due to the increased risk of uterine necrosis and infection.

Ultimately, clinical success depends not only on surgical technique, but also on the systematic implementation of updated institutional algorithms and care bundles. Simulation-based training and obstetric drills are essential to reduce suboptimal response delay during the transition between intervention stages. Given that PPH is significantly associated with psychological outcomes such as postpartum depression, it is imperative to consolidate these technical advances within a comprehensive, multidimensional care framework.

## Figures and Tables

**Table 1:** Characteristics of the studies included in the systematic review (period 2020–2024).

Author (Year)/Reference [No.]	Study Design	Sample Size (*n*)	Intervention/Evaluated Technique	Main Outcomes (Efficacy/Complications)
Gallos ID, et al. (2018/2025) [8]	Systematic Review/Meta-analysis	Various studies (multicentric)	Uterotonic Agents (Prophylaxis)	Higher efficacy of carbetocin; oxytocin is standard. Reduces PPH > 500 mL.
Ker K, et al. (2024) [14]/Sentilhes L, et al. (2015) [28]	Systematic Review/Meta-analysis	Various clinical trials	Tranexamic Acid (TXA)	Reduces death by bleeding and need for surgery; positive global effect.
Suarez S, et al. (2020) [31]/Tindell K, et al. (2013) [32]	Systematic Review/Observational Study	Multicentric/20–50 cases	Intrauterine Balloon (Bakri)	Success rates of 80–90% in control of refractory bleeding. Fewer hysterectomies.
Hofmeyr GJ (2023) [10]	Narrative Review	N/A (Novel Concepts)	Uterine Vacuum Devices (Jada)	Faster bleeding control times (minutes vs. hours).
Luo L, et al. (2021) [17]	Case Series	*n* = 2	B-Lynch + Uterine Devascularization	Higher risk of uterine necrosis and infection. Caution in combined use.
Kaya B, et al. (2015) [13]	Observational Study	*n* = 28	B-Lynch Compressive Sutures	Efficacy of 68.4% to 100%. Effective and safe alternative for atony.
Sentilhes L, et al. (2009) [29]/Loaec C, et al. (2015) [16]	Observational Study/Follow-up	*n* = 11/*n* = 40	B-Lynch (Fertility Preservation)	Does not appear to compromise subsequent fertility. Favorable obstetric outcomes.
Alonso-Burgos A, et al. (2024) [2]	Narrative Review/Case Series	*n* = Variable (Series)	Uterine Artery Embolization	High efficacy in hemodynamically stable patients. Low complication rate.
Vogel JP, et al. (2024) [33]	Systematic Review	Multicentric	‘Care Bundles’ and Protocols	Improves adherence to guidelines and reduces severe complications in PPH.
Mendez-Figueroa H, et al. (2022) [19]	Observational Study	Multicentric	Obstetric Simulation and Drills	Reduction of adverse events and improvement of care response.
Jung YW, et al. (2024) [12]	Retrospective Study (11 years)	*n* = 127 (Severe PPH)	Stepped Management Protocols	Lower complications and hysterectomy rates in centers with standardized protocols.
Ende HB, et al. (2021) [5]	Systematic Review/Meta-analysis	Multicentric	Early Identification of Risk Factors	Factors like prenatal anemia, induction, and multiparity are essential.
Sheng B, et al. (2024) [30]	Observational Study	*n* = Variable (Series)	Integral Impact (Psychological)	Significant association between PPH and postpartum depression.

Note: 13 studies were selected that met the PRISMA 2020 criteria for the period 2020–2024, evaluating key interventions and organizational factors.

## Data Availability

The data supporting the findings of this review are derived from publicly available published studies cited in the reference list. No new datasets were generated, and no analytic code was used because no quantitative meta-analysis was performed.
